# RDMkit: A research data management toolkit for life sciences

**DOI:** 10.1016/j.patter.2025.101345

**Published:** 2025-08-22

**Authors:** Pinar Alper, Flora D'Anna, Bert Droesbeke, Munazah Andrabi, Rafael Andrade Buono, Federico Bianchini, Korbinian Bösl, Ishwar Chandramouliswaran, Martin Cook, Daniel Faria, Nazeefa Fatima, Rob Hooft, Niclas Jareborg, Mijke Jetten, Diana Pilvar, Gil Poires-Oliveira, Marina Popleteeva, Laura Portell-Silva, Jan Slifka, Marek Suchánek, Celia van Gelder, Danielle Welter, Ulrike Wittig, Frederik Coppens, Carole Goble

**Affiliations:** 1 Luxembourg National Data Service, PNED GIE, 4362 Esch-sur-Alzette, Luxembourg; 2 VIB Data Core, VIB Technologies, 75 9052 Ghent, Belgium; 3 The University of Manchester, Oxford Road, Manchester M13 9PL, UK; 4 ELIXIR Hub, Wellcome Trust Genome Campus, Hinxton, Cambridge CB10 1SD, UK; 5 INESC-ID, IST, University of Lisbon, Lisbon, Portugal; 6 Barcelona Supercomputing Center, Eusebi Guell Square 1-3, 08034 Barcelona, Spain; 7 Centre for Bioinformatics, University of Oslo, Oslo, Norway; 8 The Arctic University of Norway, Tromsø, Norway; 9 Heidelberg Institute for Theoretical Studies, 69118 Heidelberg, Germany; 10 Department of Informatics Computational Biology Unit, University of Bergen, Bergen, Norway; 11 Stichting Health-RI, Jaarbeursplein 6, 3521 AL Utrecht, the Netherlands; 12 Office of Data Science Strategy, National Institutes of Health, Rockville Pike, Bethesda, MD 20892, USA; 13 National Bioinformatics Infrastructure, Department of Cell and Molecular Biology, Uppsala University, Uppsala, Sweden; 14 ELIXIR Estonia, University of Tartu, Tartu, Estonia; 15 Luxembourg Institute of Health, 1 A-B Rue Thomas Edison, 1445 Strassen, Luxembourg; 16 Department of Chemistry, NOVA School of Science and Technology, NOVA University, Caparica, 2829-516 Lisbon, Portugal; 17 Czech Technical University in Prague, Thakurova 9, 160 00 Prague, Czech Republic

**Keywords:** data lifecycle, guidelines, FAIR, RDM community, tool assemblies

## Abstract

The rise of data-driven scientific investigations has made research data management (RDM) essential for good scientific practice. Implementing RDM is a complex challenge for research communities, infrastructures, and host organizations. Generic RDM guidelines often do not address practical questions, and disciplinary best practices can be overwhelming without proper context. Once guidelines are established, expanding their reach and keeping them up to date is challenging. The RDMkit is an open community-led resource designed as a gateway to reach the wealth of RDM knowledge, tools, training, and resources in life sciences. The RDMkit provides best-practice guidelines on common RDM tasks expected of data stewards and researchers, specific data management challenges and solutions from life science domains, and tool assemblies showcasing holistic solutions to support the research data life cycle. Built on a reusable open infrastructure, the RDMkit allows organizations to create their own guidelines using it as a blueprint.

## Highlights

• RDMkit is a research data management (RDM) knowledge resource for the life sciences
• Offers practical guidelines with links to RDM tools, training, and knowledge
• Empowers communities with an open, adaptable framework to build disciplinary guidelines

## Graphical abstract

**Figure:**
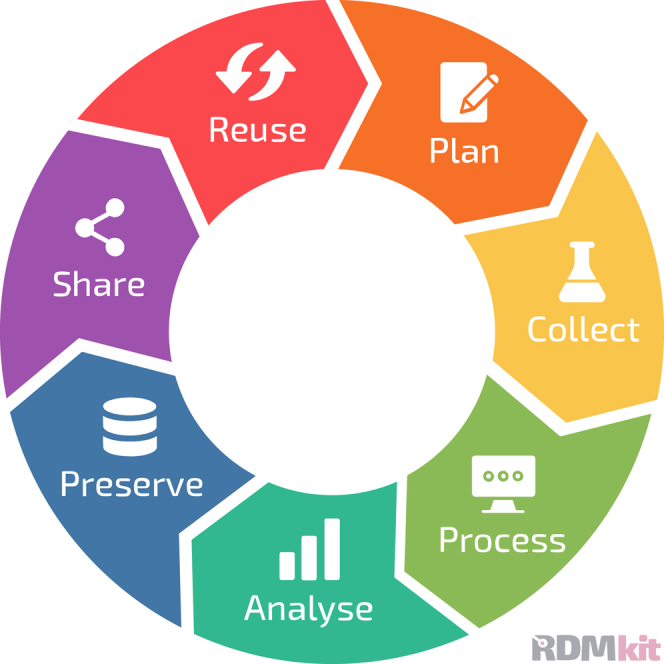

## Teaser

The rise of data-driven science has made research data management (RDM) essential, yet implementing it remains complex due to diverse disciplinary needs, limited tailored guidance, and gaps in training and knowledge among researchers. The RDMkit is a community-led, open resource that addresses these challenges by offering practical, domain-specific RDM guidance along with links to relevant tools, training, and knowledge for life sciences. Built on a reusable infrastructure, RDMkit supports the full data life cycle and enables organizations and communities to establish their own guidelines.

## Introduction

Research data management (RDM) refers to mindful handling of data during and after research, including its collection, organization, storage, documentation, sharing, and archiving.^1^ Within the last decade, RDM has become part of good scientific practice that is encouraged and expected of researchers and, in tandem, an implementation challenge for research-performing organizations.^2^ On the one hand, organizations recognize that RDM enhances the rigor and reproducibility of scientific research and ensures legal and ethical compliance in research activities. It is a keystone for implementing funders’ open science policies, which call for findable, accessible, interoperable, and reusable (FAIR) data that are shared as widely as possible to foster global research efficiency and collaboration.^3^ On the other hand, researchers benefit from systematic RDM to improve their own productivity, enable sophisticated data processing and modern AI methods, ease collaboration with peers, and smooth pathways to publication.^4^ Despite these strong drivers, practical RDM implementation remains a multifaceted challenge for researchers, their research organizations, and the multinational research infrastructures that they work within.^5^

Adopting RDM requires a change in the culture of both research and research support.^5^^,^^6^ Organizations and research infrastructures must equip researchers with RDM tools, resources, training, and expertise. In addition, they should offer clear descriptions of the fundamental practices expected in RDM, including adherence to FAIR principles, open science policies, and ethical and legal requirements. Likewise, researchers must cultivate a deep understanding of the research data life cycle and seamlessly integrate the necessary data management practices into their research process in a structured and timely manner.^7^ To foster this change, funders ask for data management plans (DMPs) as funding prerequisites, and to aid organizations and researchers in the process, they provide DMP templates and guidelines.^8^^,^^9^ As funders support research in diverse disciplines, these guidelines are broad, covering universal, domain-independent data management principles. As such, while these guidelines are essential for policy compliance, they do not address the myriad of RDM questions researchers ask, such as “How do DMP requirements translate to my domain or data type?” “What are the common RDM practices and solutions for my discipline?” “Which national resources are available to me?” “When should I use which tool?” and “Can I use tools in combination?” Without answers to these questions, generic DMP guidelines fall short of a transformative effect.^10^

On the flip side, in scientific domains where data-driven discovery is commonplace, such as the life sciences, there is advanced community data literacy and a myriad of software, databases, metadata standards, and templates. For newcomers in these domains, the sheer number of tools and resources available can be overwhelming, especially without introductory guidelines, context, and clear directions. Moreover, the best practices for data management developed in (sub)communities often remain implicit. Even when documented, they are seldom framed in terms of how they align with the research data life cycle, the funder DMP requirements, and FAIR principles.

Enterprise data management frameworks use a clear identification of the stakeholders involved, along with their roles and responsibilities.^11^ However, typically, this is not the case in RDM practice in the academic setting^12^: researchers still have the main responsibility for planning and executing data management, supported by various stakeholders, including data stewards, principal investigators, lab and facility managers, research software engineers, trainers, and funders.^13^ These support roles are not well defined in existing RDM guidelines. Collecting, managing, and sharing an ever-growing amount of data in line with state-of-the-art practices, as well as adhering to relevant policies and regulations, demand specialized skills and knowledge. This places a heavy burden on researchers, who must learn and adopt both generic and disciplinary best practices.^6^

Research institutions are stepping up to the challenge by providing RDM guidelines and training and hiring data stewards to inform and assist researchers.^14^ Such institutional support is just emerging; researchers and data stewards also receive similar support at the national or international level from networks and research infrastructures.^15^^,^^16^^,^^17^ As data stewardship support is established, the foremost challenge is scaling in order to close the RDM knowledge gap. To illustrate from life science, against the estimated 500,000 life scientists in Europe, the newly established community of data management practitioners has members of a few hundred.^18^ Additionally, the specificity and applicability of RDM practices vary across communities. For instance, while all human-subject research must adhere to data protection regulations regarding data documentation, human genomics research requires specific solutions to mitigate reidentification risks. As such, it is challenging for data stewards to simultaneously maintain cross-disciplinary and discipline-specific guidelines. Once a guideline is generated, the next problem that arises is to keep its content fresh. Research communities constantly develop new tools and resources to optimize their data use, and keeping guidelines up to date against the dynamic scientific resource landscape is another challenge that data stewards face.

In 2020, ELIXIR,^17^ the European research infrastructure for life science data, began efforts to standardize data management across its partnering organizations. Our preparatory landscape analysis highlighted all these RDM challenges.^19^ Numerous software tools, databases, policies, standards, databases, and training programs were indexed and described in dedicated registries,^20^ but they lacked context on how they could be integrated into solutions for general or discipline-specific RDM problems. Emergent specialist RDM resources, such as the Data Stewardship Wizard (DSW) for data management planning^21^ and the FAIR Cookbook for data FAIRification,^22^ aimed to capture the expertise of our scarce data managers and stewards but were disconnected from one another.

The RDMkit (https://rdmkit.elixir-europe.org), launched in 2021, is our response to address the RDM challenges in the life sciences. RDMkit is a community-driven web toolkit for researchers and data stewards and is the common gateway to ELIXIR’s RDM resource ecosystem. RDMkit guidelines represent a combined effort of RDM practitioners across 22 countries within and beyond Europe. RDMkit contributors include other research infrastructures, such as the Biobanking and BioMolecular Resources Research Infrastructure (BBMRI) (https://www.bbmri-eric.eu), Instruct (https://instruct-eric.org), Euro-BioImaging (https://www.eurobioimaging.eu/how-to-access/how-to-acknowledge-us/), and the US National Institutes of Health (NIH) (https://www.nih.gov). RDMkit has become a recognized resource of life science RDM information. The RDMkit approach to guideline development has been replicated, and its infrastructure has been reused by various communities within and beyond life science.

In this paper, we outline the three core components of RDMkit: (1) a live, up-to-date resource containing generic and disciplinary RDM guidelines for the life sciences; (2) an open community with well-defined participatory processes for content creation and maintenance; and (3) a reusable infrastructure that can be adopted to develop similar guidelines in other scientific domains. We also discuss RDMkit adoption, use, and sustainability plans, as well as its ongoing impact on the global scientific community.

## Results

### RDMkit: A knowledge resource developed by and for RDM practitioners and researchers

RDMkit is an open, community-driven knowledge resource providing good data management practices—in line with the FAIR principles—for the life sciences. RDMkit has guidelines, practical information, and pointers to other RDM resources, and it organizes its advice in a way that follows the life cycle of data from the start to the end of research projects. RDMkit was created and is maintained through open collaboration by the RDMkit community, composed of RDM practitioners and researchers from several domains. RDMkit is based on an open-source, reusable technological core, which allows communities, initiatives, and projects to easily deploy online guidelines.

The main goal of RDMkit is to provide information to researchers so that they can plan and execute their data management using state-of-the-art tools and current best practices. RDMkit content is intended to close the RDM guidance gap (1) by providing context to the tools, showing when to use them in a project and for which RDM activity and by whom they should be used, as well as how various communities have combined tools to provide custom data management solutions; (2) by placing itself centrally with bidirectional connections to other RDM knowledge resources, making it possible for researchers to navigate the RDM knowledge space without getting lost; and (3) by providing live content looked after by an active community using well-defined processes to ensure that the guidance stays up to date.

The key target audience of RDMkit is life science researchers, such as biologists and bioinformaticians and data stewards. For researchers, RDMkit is a one-stop shop for information, advice, and signposting to RDM know-how, resources, examples, and best practices. Simultaneously, RDMkit aims to address the needs of professionals in RDM, such as data stewards with different domain areas, responsibilities, and tasks, which directly support researchers by providing advice, training, RDM services, tools, and infrastructures. Data stewards can use RDMkit to complement their expertise, resources, and training material. Funding agencies and policymakers can benefit from the RDMkit by including it in their DMP guidelines and exemplifying how life science communities have approached implementing open-science requirements and FAIR principles with state-of-the-art tools and resources.

Since its launch in March 2021, 218 contributors have authored 134 guidelines in RDMkit. Nineteen of those guidelines target specific domains of life science. The RDMkit guidelines refer to a total of 673 data management tools and resources.

### RDMkit content structure and user flow

**Figure 1:**
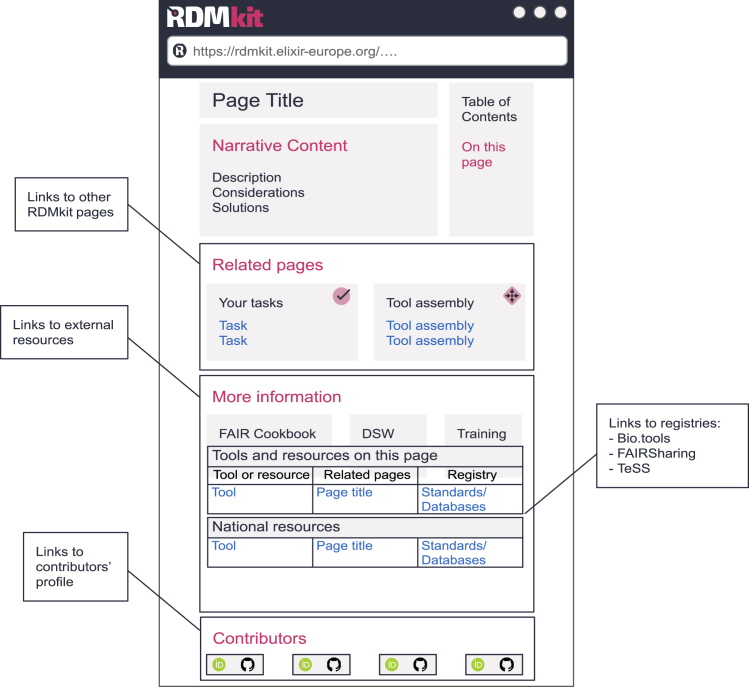
RDMkit page anatomy Wireframe illustrating the various segments in an RDMkit page.

RDMkit offers its guidelines in six major sections: “Data life cycle,” “Your role,” “Your domain,” “Your tasks,” “Tool assembly,” and “National resources.” The details of the sections are provided in Table 1. Each of the sections is organized in various topics, allowing swift navigation to relevant content. Per topic, the guidance is presented in a single webpage, referred to as an “RDMkit page.” RDMkit pages are cross-referenced and highly structured with multiple segments; Figure 1 illustrates the anatomy of the RDMkit page. Per page, guidance is provided as a combination of a narrative segment, a “Related pages” segment that contains links to other RDMkit pages, and a “More information” segment containing links to external knowledge and information resources and to a table of tools and resources explicitly mentioned in the narrative segment.

**Table 1:** RDMkit sections containing best-practice guidelines

**Data life cycle—an introduction to RDM**
It is customary to introduce RDM to beginners by organizing information along the research data life cycle (see “related work”). The RDMkit life cycle involves the stages of data management planning, data collection, processing, analysis, preservation, sharing, and reuse. For each stage, the RDMkit introduces what the stage involves, why it is essential, and what the key considerations are for that stage.
**Your tasks—solutions to common RDM problems**
Throughout research projects, researchers and data stewards collaborate to solve common data management issues, such as writing data management plans, calculating storage costs, and licensing data. RDMkit identifies these as generic tasks and offers practical approaches and solutions. The RDMkit page template guides authors to structure content around a practical problem, lists key considerations for addressing the task, and provides possible solutions with references to relevant tools and resources.
**Your domain—solutions tailored to life science domains**
Specific types of life science data present unique challenges in RDM due to factors such as data size, sensitivity, structure, or analysis methods. Over the years, life sciences communities have developed best practices for managing their data. This section of RDMkit offers a collection of RDM guidance from various scientific communities, such as plant sciences, bioimaging, biomolecular simulations, epitranscriptomics, and human genome/phenome and health data. An example of how general (“Your tasks”) and domain-specific (“Your domain”) guidance complement each other is reflected in ontology recommendations: “Your task” directs users to general ontology portals, while “Plant sciences” specifies the crop ontology, guiding users to the most relevant resources based on their specific questions. This is just one example—this logic applies across all guidelines.
**Your role—useful information for your professional role**
RDM is a collaborative effort that involves various research and support roles. RDMkit outlines the responsibilities of all relevant roles, including researchers, data stewards, principal investigators, policymakers, trainers, and research software engineers.^23^ For each role, it provides the most relevant guidance and links to additional RDM resources and training.
**Tool assembly—use it or get inspired**
Institutes, national ELIXIR nodes, and scientific communities have, over the years, developed tools that support data management throughout the data life cycle. These solutions are called “tool assemblies” in RDMkit, and their role as guidance is 2-fold. First, they inform users in specific countries or disciplines about available assemblies on national or community platforms. Second, they present open-source, reusable tool assemblies in the RDM context, inspiring other users, institutions, or infrastructure providers to adopt these assemblies, partially or fully.
**National resources—country-specific information**
While many data management challenges are universal, national policies, legal frameworks, and institutional requirements must be considered when implementing DMPs. The “National resources” section in RDMkit complements the international guidance by providing country-specific resources, relevant funder policies on open science and data management, regulations related to data ethics, and information on available national infrastructure for researchers. These pages also highlight RDMkit-listed tools developed within each country.

For the narrative segment, RDMkit offers lightweight templates to guide page authors. The guidance narrative outlines key considerations—important aspects to consider before making decisions—and solutions on the RDM topic. These solutions include recommended approaches and references to various software tools and resources, such as policies, standards, and databases. The RDMkit page narrative provides the crucial context in which tools and resources could be used and helps one select the appropriate ones.

The “Related pages” segment suggests links to other RDMkit pages on relevant topics. The “More information” segment links to external resources, specifically the online training material, the FAIR Cookbook recipes, and the questionnaire-style DSW guidance relevant to the page topic. The “Tools and resources” table at the bottom of each page provides a page-level summary with links to related training and further information that can be found for each listed tool or resource in life science registries. Finally, in the “Contributors” segment, those who have created, reviewed, and edited content are acknowledged by listing their names and online profiles, such as GitHub or ORCID.

**Figure 2:**
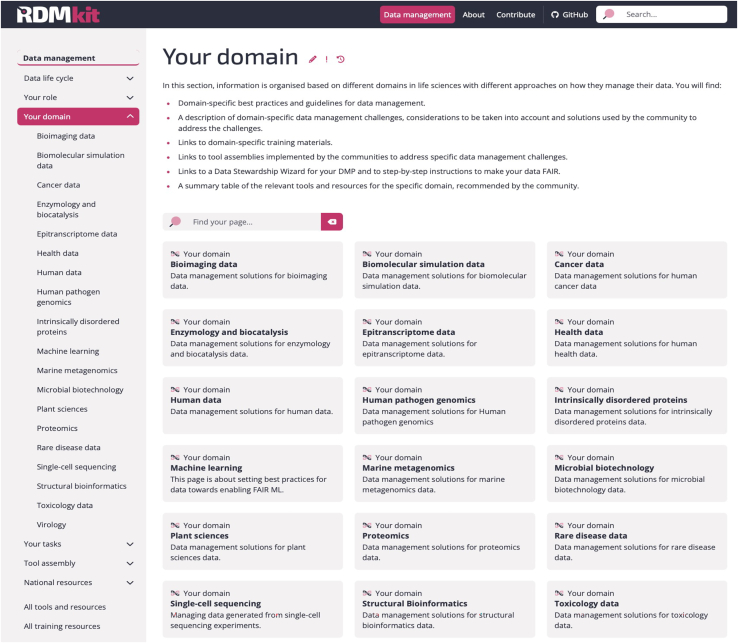
An example RDMkit page Screenshot displaying the top and left-hand navigation menus and the section overview for “Your domain” pages.

**Figure 3:**
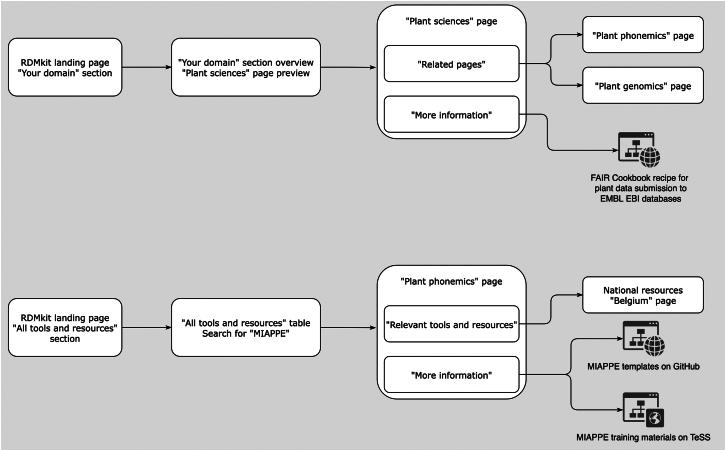
Example site user flows for a plant biologist seeking guidance in RDMkit The top part illustrates a user’s exploration of “Plant sciences” pages. From the landing page, they navigate to the “Your domain” section and select the “Plant sciences” page. There, they find two “Tool assemblies” pages, “Plant genomics” and “Plant phenomics,” detailing tool combinations for plant data management. In the “More information” segment, they discover a FAIR Cookbook recipe for submitting plant genomic and genetic variation data to EMBL-EBI (https://www.ebi.ac.uk) databases. The user bookmarks the “Plant sciences” page for future reference. The bottom part illustrates a user’s discovery of resources on the Minimal Information about a Plant Phenotyping Experiment (MIAPPE) standard. The user seeks resources to self-train for creating metadata for a plant phenotyping experiment. They navigate to the “All tools and resources” section and search for MIAPPE in the tools table. Finding it mentioned on the “Plant phenomics” page, they navigate there, and in the “More information” segment, they find a TeSS shortcut to MIAPPE training materials and spreadsheet templates with example data. Under “National resources,” they discover that Belgium hosts the PIPPA (https://pippa.psb.ugent.be) tool for publishing raw plant phenotyping datasets with MIAPPE metadata. The user bookmarks the “Plant phenomics” page for future reference.

Each RDMkit section has an overview page providing links to the pages within; an example screenshot for the “Your domain” section is provided in Figure 2. In addition to the data management guidelines, the RDMkit site provides the “Contribute” and the “About” sections. Navigating across these sections is possible with a top-level menu; meanwhile, navigation within each section is possible with the left-hand side menu, both illustrated in Figure 2. All tools, resources, and training mentioned on all RDMkit pages are available in two aggregated tables, which can also be reached via the left-hand navigation menu of the website; see Figure 2. These segments are based on page metadata and are automatically generated; details are described in the methods. Figure 3 illustrates how users can navigate within and beyond the RDMkit website using these links.

### Integrations with other RDM knowledge resources

One of the objectives of RDMkit is to help readers navigate the plethora of RDM knowledge available to life scientists. RDMkit achieves this by complementing its thematic guidelines with pointers to further information in other resources in the FAIR life science knowledge ecosystem. Currently, the following resources are integrated with RDMkit:

(1) DSW^21^ is a tool that leads users through decision trees to help them choose the right tools, resources, and practices when planning their data management.
(2) FAIR Cookbook^22^ is an online collection of “recipes,” which are step-by-step instructions for data FAIRification and management.
(3) The TeSS Training Portal^24^ is ELIXIR’s registry of life science training events and materials aggregated from the national ELIXIR nodes and third-party providers.
(4) bio.tools^20^ is a registry of bioinformatics software resources, including biological databases and software tools.
(5) FAIRsharing^25^ is a registry of scientific data and metadata standards, databases, and data policies.

Integrations in RDMkit are not simple links but the result of both conceptual and syntactic curation by contributors, editors, and collaboration with the managers of external resources such as TeSS, DSW, and FAIR Cookbook. RDMkit, along with DSW and FAIR Cookbook, is an ELIXIR Recommended Interoperability Resource, and its integrations with ELIXIR resources are overseen by the ELIXIR Interoperability Platform to ensure compliance with best-practice standards.

Integrations with DSW are based on its application programming interface (API), while machine-actionable metadata—specifically, YAML files and structured front matter in both RDMkit and FAIR Cookbook markdown pages—support automated display of connections. This combined use of APIs and structured metadata enables flexible, scalable, and low-maintenance implementation (see methods for details on integration with each resource). Decisions on new integrations are made collaboratively by the RDMkit editors and community, guided by the relevance and usefulness of additional resources.

Integrated resources are reachable from RDMkit in different ways. Links to DSW, the FAIR Cookbook, and TeSS can be found on each page, pointing to more tailored and practical guidance or training on the page topic. Links to bio.tools, FAIRsharing, and TeSS can be found in the tools table, pointing to provider-supplied, curated, up-to-date information on each tool.

#### Interactive and task-specific guidance

**Figure 4:**
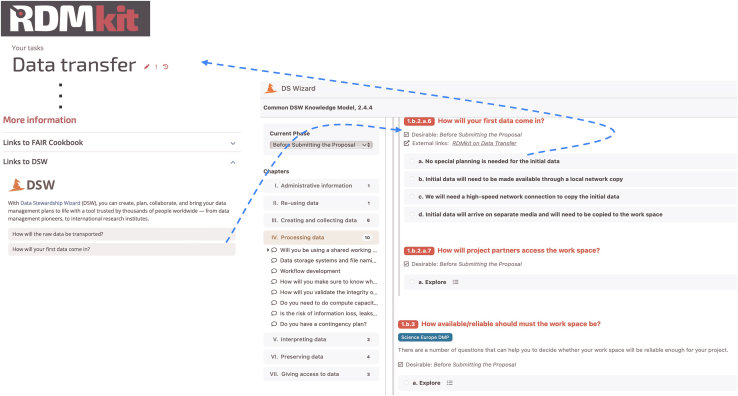
Screenshot displaying Data Stewardship Wizard links on the RDMkit “Data transfer” page Users of RDMkit are referred to the DSW to launch an interactive guidance session and make context-dependent decisions between alternative RDM solutions. In key places in the knowledge model, DSW users are referred to RDMkit for background information and overviews.

In RDMkit we provide links to two comprehensive knowledge models on data management that are implemented in DSW: one for research data in general and another containing small additions and adaptations specific to life science data. The decision trees provide an alternative way to choose the right tools, resources, and practices when planning data management. Parallel to developing RDMkit guidelines, we extended these knowledge models to include domain and task-specific decisions and guidance. The updated knowledge models are part of the generally available DSW installation package and are also available in a DSW deployment integrated with RDMkit. The DSW and RDMkit are bidirectionally linked (see Figure 4).

#### Discovering life science training

**Figure 5:**
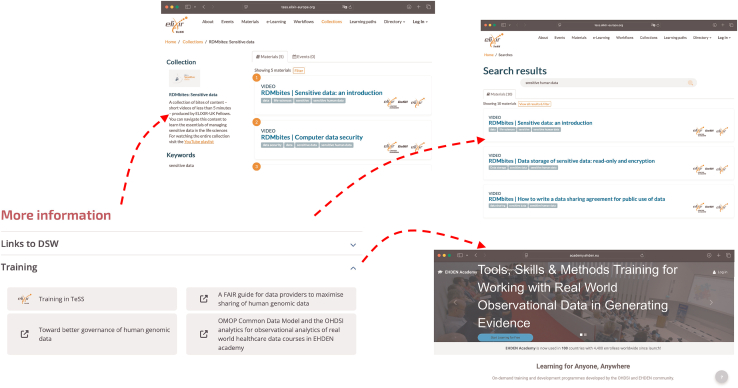
Screenshot displaying the “Training” segment on the RDMkit “Human data” page Training links can be toward TeSS training collections, TeSS searches, and other external training resources.

Training is a key element, which, combined with RDM best practices and tools, results in increased data management and stewardship capacity for RDMkit users. TeSS integration allows RDMkit users to discover training events and materials relevant to a particular RDM topic, tool, or resource. The integration is unidirectional from RDMkit to TeSS, where the user is referred to a current TeSS search with search keywords that characterize the RDMkit page topic (see Figure 5). The scope of TeSS is life science training in general. To increase TeSS coverage on RDM training and to enable findability via RDMkit, RDM training providers from various ELIXIR nodes curated TeSS records and verified that they appear in RDMkit-directed searches.

#### Taking specific data management steps

**Figure 6:**
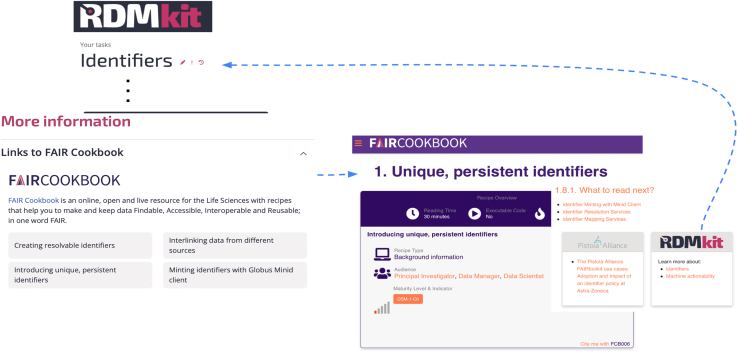
Screenshot displaying linked FAIR Cookbook recipes on the RDMkit “Identifiers” page and the respective backlinks RDMkit refers to page-relevant practical RDM recipes in the cookbook, which in return refers to RDMkit for overviews and background information.

FAIR Cookbook recipes range from step-by-step instructions for daily RDM tasks to information on FAIR implementation technology and best practices. RDMkit and FAIR Cookbook are bidirectionally connected (see Figure 6). We established a joint RDMkit-FAIR Cookbook editorial board to oversee cross-referencing between the sites (described in the methods section).

### The RDMkit website theme for deploying other guidelines

The technological core of RDMkit is based on an open-source, reusable website template, the ELIXIR Toolkit Theme (ETT). The theme abstracts the functionality of the RDMkit site and makes it available for others to create a similar site with their own content. The theme is open source and has the permissive MIT use license. Several projects and initiatives have adopted the RDMkit site theme, as described in the “RDMkit usage and uptake” section.

### The RDMkit community and processes

RDMkit processes are based on open-source community practices. The content is CC-BY licensed and, once created, is curated and maintained primarily by the RDMkit editors. No single contributor or editor owns the content; it is a living resource continually improved by the RDMkit community. The contribution process includes virtual events for online and focus groups for offline content development. RDMkit editors facilitate content development and ensure the content reflects author consensus on any topic. To help contributors, the RDMkit website provides extensive documentation on the operational processes (described in the methods section).

### RDMkit usage and uptake

The impact of RDMkit can be evaluated based on the contributions from various communities and organizations, as well as the recommendations from funders. Shortly after its release in 2021, the RDMkit was endorsed in the European Commission’s Horizon Europe Programme Guide^26^ and the ERC Guideline^27^ as a “resource for Data Management guidelines and good practices for the Life Sciences.” As the initial driving force behind RDMkit, the national ELIXIR nodes continue to play a key role in its adoption across Europe. Numerous ELIXIR nodes, communities, platforms, and EU projects have embraced RDMkit and have made it a central resource for the new ELIXIR RDM community. RDMkit is embedded in the nodes’ community outreach, training, and local guidelines^28^ and is being included in national funders’ recommendations.^29^

The recognition of RDMkit goes beyond EU funding bodies and ELIXIR nodes. Other European Research Infrastructure providers, such as EuroBioImaging (https://www.eurobioimaging.eu) and BioExcel Centre of Excellence for Molecular Modeling (https://bioexcel.eu), have contributed to specialist pages. The RDMkit website is visited by users from around the globe, including the Australian, Asian, African, and South American continents, as well as North America and Europe. RDMkit is referenced by more than 434 web domains, including websites of several funding agencies, ELIXIR nodes, universities, and research institutes. The largest group of visitors to the RDMkit site is from the United States, a direct result of RDMkit being referred to in guidelines of academic and research institutions in this country. The NIH supports RDMkit: the Office of Data Science Strategy (ODSS) is represented on the RDMkit editorial board.

Aside from content, RDMkit’s site infrastructure and process have garnered significant adoption. The RDMkit site theme has been adopted by the Australian BioCommons (https://www.biocommons.org.au), WorkflowHub (https://workflowhub.eu), the Infectious Diseases Toolkit (https://www.infectious-diseases-toolkit.org), the FAIRDOM Consortium website (https://fair-dom.org), the 1+ Million Genomes Trust Framework (https://framework.onemilliongenomes.eu), and the Research Software Quality Kit (https://everse.software/services/rsqkit/). The adoption of RDMkit has extended beyond life sciences; RDMkit has inspired the NFDI4Chem knowledge base (https://knowledgebase.nfdi4chem.de/knowledge_base/), which delivers RDM guidance for chemistry. The NFDI4Ing Engineering Sciences Community (https://nfdi4ing.de) has included RDMkit in its shortlist of the best RDM solutions in 2021.

## Discussion

### Related work

While there is currently no theoretical model for RDM, the research data life cycle is a commonly used framework to organize RDM knowledge. The research data life cycle typically incorporates the stages of data management planning, data collection, processing, analysis, preservation, publishing, and sharing, finally coming to a full circle with data reuse. While there are several research data life cycles available online, the UK JISC^30^ and the UK Data Archive^31^ models are well documented, and as such, they have served as the basis for the RDMkit’s data life cycle.

For researchers seeking knowledge about RDM, universities, university libraries, and funders provide the first line of support through online RDM guidelines. These guidelines outline the expectations of research host institutions and/or funders on data management and sharing, and as such, they are important information resources for researchers to achieve data policy compliance. Furthermore, these guides act as necessary signposts, as they list resources available to researchers to support their journey throughout the research data life cycle. As institutions or funders support research from a multitude of domains, their guidelines are often generic, covering domain-agnostic data management principles. As such, their advice is complementary to discipline-specific resources such as the RDMkit.

The Turing Way^32^ is a resource developed by a grassroots international community as an open-source handbook on reproducible data science. The Turing Way covers data management in a basic and domain-agnostic way; it provides the essential definitions of research data, FAIR principles, and DMPs as well as good data handling practices at various stages of data science projects, from finding data to data cleaning, curation, analysis, visualization and sharing. RDMkit’s participatory processes were inspired by the Turing Way, particularly its community-driven nature and its approach to content co-creation via dedicated events.

### RDMkit present and future

Making good data management an integral part of research is a multipronged effort that includes funders, research infrastructures, RDM practitioners, and researchers. RDMkit is a resource developed by and for these stakeholders, and, with its guidelines, community, and reusable infrastructure, it provides multipillared support for RDM in the life sciences.

From the outset, we positioned RDMkit as a venue for specific, illustrative, and tangible guidance. The strong interest in communities and the ever-growing list of RDMkit “Domain,” “Tool assembly,” and “National resource” pages are clear evidence that such guidance was acutely needed. We see RDMkit’s role as a navigational aid as critically important, especially for assisting researchers on the RDM learning curve. Built on domain-agnostic foundational principles, consolidated in its “Data life cycle” and “Your tasks” pages, RDMkit aids data stewards in providing solutions to RDM inquiries, making it a current and valuable resource for data stewards in their user support roles. RDMkit also acts as a central signpost, allowing users to navigate the landscape of RDM resources, which is enabled by integrations with DSW, TeSS, FAIR Cookbook, bio.tools, and FAIRsharing. Another vital component that led to the successful development of RDMkit and its community was the strong, supportive leadership. Leaders were committed not only to the project’s success but also to supporting the people involved. The project was managed as an egalitarian effort, giving all stakeholders an equal voice in shaping the toolkit. The leadership set clear goals while allowing members the freedom to align the project with their vision and the community’s needs. Additionally, dedicated funding helped maintain momentum and keep all partners engaged and focused.

RDMkit is designed and developed with sustainability in mind. The site technology is intentionally kept simple, and the site infrastructure is kept separate from the content as a modular and reusable site theme. We established a lightweight, open process to effectively harness the community’s collective expertise. Another approach taken toward sustainability was the adoption by ELIXIR nodes. To date, the national ELIXIR nodes of Belgium, Norway, Sweden, and the UK have included the RDMkit content and infrastructure in their service delivery plans, providing a roadmap and sustainability resources. This adoption ensures that RDMkit usage extends to multiple grants, projects, and collaborations related to research infrastructures. In addition, nodes leverage RDMkit as a resource for their institutes and universities, further embedding its utility in institutional frameworks and enhancing its role in the broader RDM landscape. The broad adoption of the RDMkit site theme in several nodes and European projects calls for a governance system to sustain this tool.

The true power and potential of RDMkit lies in its community. The successful development of the RDMkit community was primarily driven by stakeholders’ commitment to building sustainable solutions for the scaling challenges in RDM. This project united a motivated community with a shared purpose of addressing RDM challenges. The enthusiasm with which the community engaged in building the RDMkit highlighted a shared sense of urgency in tackling RDM issues. We recognized that community is not merely a by-product of a project; it is, in fact, essential to its success. Leveraging existing tools, registries, and resources meticulously built by the life science community, we tapped into established networks. The ELIXIR networks helped assemble a passionate and dedicated team. We established a lightweight, open process to effectively harness the community’s collective expertise. RDMkit’s participatory processes lower the barriers to contributions so that it is familiar to the broadest possible contributors. Authoring and review processes are open, all content is open sourced and version controlled, and contributor credit is central. All future sustainability plans include the sustainability of the successful RDMkit community.

The challenges that the RDMkit’s success brings are scaling up its content, keeping it up to date, and keeping the contributing communities engaged. To address these, we are employing three main sustainability strategies. First is content coordination and the maintenance of the integrations with ELIXIR resources, such as FAIR Cookbook, TeSS, and DSW. The ELIXIR Research Data Management Community^18^ plays a key role as the main content provider for all mentioned resources and their integrations. In a dedicated project, the ELIXIR RDM community is mapping the possible user journeys across ELIXIR RDM resources, some of which were illustrated in this paper. Feedback from these user journeys and the RDM community will be critical in validating and improving the signpost function of RDMkit in the ELIXIR landscape. Second is extending RDMkit utilization across projects and infrastructures to expand its network, foster community building, and attract individuals and organizations to contribute. The widespread adoption of RDMkit by nodes and its integration into various projects, research infrastructures, and institutions create broader networks of engaged individuals who can actively contribute to the evolution and sustainability of RDMkit. This growing community provides a foundation for the RDMkit Club, a strategic and decision-oriented group where members can shape the toolkit’s progress by proposing new strategies for expansion, applications, and long-term sustainability. At the same time, individuals from these networks can contribute in a more practical capacity by becoming RDMkit contributors, enriching the platform with new content and resources. The RDMkit editorial board oversees the contribution process, ensuring quality and consistency while remaining open to new members, further strengthening the toolkit’s inclusivity and collaborative spirit. Together, these pathways foster a dynamic and sustainable ecosystem that drives the continuous improvement of RDMkit and ensures its relevance in the ever-evolving RDM landscape. Third is establishing a governance system, called the RDMkit Alliance, as a forum to allow us to get organized with how individuals and organizations across the globe can contribute, how they can reuse the RDMkit’s content on its underlying platform, and on its processes and people structures. For the cases where communities will need their own RDMkit instances, the RDMkit Alliance will play a critical role in enabling all stakeholders to engage and be part of the continuing venture.

Together, these pillars—community building, widespread adoption, and the establishment of the RDMkit Alliance—lay the foundation for a unified, global effort that empowers stakeholders to collaboratively shape the future of RDM, ensuring that RDMkit remains a vital, evolving, and impactful resource for the future.

## Methods

Five key decisions shaped the RDMkit approach: (1) we collected interviews and user stories to base content on user needs. (2) We kept the technology stack simple for usability and sustainability. (3) To keep within a manageable scope, we identified up front, with a scope-setting exercise, what the RDMkit will and will not provide. The scope of RDMkit guidelines is kept intentionally focused and concise, integrating existing resources. (4) We established continual community-based content development to accommodate emerging RDM insights, policies, and solutions. (5) We followed best practices from software and web development to ensure quality, security, accessibility, and sustainability. The following sections outline the RDMkit methodology.

### User requirement gathering

RDMkit’s user requirements process was based on the personas participating in the research data life cycle and their user stories. We initially focused on two personas: researchers and data stewards. The user stories were structured using the commonly known template of who/what/why, e.g., “As a researcher, I want to find the appropriate tools and pipelines so I can process my marine datasets.” User stories were then translated into the scope and technical requirements of the website. We analyzed existing RDM toolkits for features and sketched wireframe storyboards for the initial RDMkit designs.

From the outset, our goal was to address data management needs across the entire research data life cycle. To support this, we developed the “Data life cycle” section in RDMkit, designed to guide users through each phase of the RDM journey. The main stages were initially based on established models and then refined using the contributors’ practical experience and expertise.

The rest of the RDMkit’s structure and layout were informed by a systematic process of identifying the user requirements of personas involved in the research data life cycle as well as analyzing their associated user stories. The objective was to elicit, extract, and prioritize key requirement feature sets in order to design a framework that would ensure the resource is practical, relevant, and valuable to its intended users.

Each persona represents the goals and behaviors of a hypothesized user group, synthesized from user interviews and the collective experience of collaborators. These personas possess diverse skill sets and occupy different positions within the research data life cycle, often fulfilling multiple roles, e.g., a researcher may act as a data creator, data user, data analyst, or data curator. The “Your role” section was built around these personas, initially focusing on researchers and data stewards and later expanding to include policymakers, principal investigators (PIs), research software engineers (RSEs), and trainers. Recognizing the importance of domain-specific expertise, we collected user stories from three research domains: marine metagenomics, human data, and plant sciences. These stories followed a standard who/what/why format (e.g., “As a researcher, I want to find the appropriate tools and pipelines so I can process my datasets.”) and informed the development of the “Your domain” section.

The “Your task” section was created to address the need for structuring RDM guidelines around common activities that apply across domains in the life sciences, making it easier for users to find relevant guidance regardless of their specific field. The “Tools” section was shaped by the recognition that users need context to understand when and why to use a particular resource—simply listing tool names is not effective. “Tool assemblies” were introduced in direct response to user feedback requesting practical, real-world examples of how tools and practices are combined in specific domains or organizations. Finally, the “National resources” section was developed to answer a recurring user question: “What’s useful for me?” By pointing to relevant resources at the national or institutional level, the toolkit helps users connect general recommendations to their local context.

Most RDMkit pages are created during virtual events called content-a-thons. These events are guided by a content gap analysis conducted by editors who consider feedback from the site’s feedback form, issue tracker, and input from various life science projects and communities. During content-a-thons, the site’s structure is also reviewed. Editors may reorganize sections, merging or splitting pages as needed to improve navigation and coherence. In addition to content development, the RDMkit site has undergone a user experience (UX) assessment, and the resulting UX improvement suggestions were implemented during the Biohackathon Europe 2021 (https://elixir-europe.org/events/biohackathon-europe-2021).

### Website technology and development

We use GitHub (https://github.com) for the RDMkit source/content control and site hosting. Several factors have informed our choice of GitHub. First, GitHub has a wide user base, including RDMkit contributors. GitHub by design enforces good collaborative processes for distributed contribution and facilitates the creation of additional content by providing a graphical user interface (GUI). Various GitHub features, such as suggesting changes to a repository, reviewing suggested changes, automatic assignment of reviews to repository section editors, changing history, code of conduct, and issue templates and labels, have shaped RDMkit’s contribution and editorial processes. We use GitHub’s repository maintenance feature to automate deployments and maintain site data and configurations. Finally, GitHub as a site-hosting platform is free, requires little or no maintenance, and is widely used, contributing to the sustainability of RDMkit.

RDMkit makes use of Jekyll, a static website generator, with the ETT on top. The theme is open source, version managed, and well documented. ETT follows best practices in web development by supporting security via static website content, standardizing accessibility by following the standard Web Content Accessibility Guidelines^33^ and achieving privacy by minimizing the collection of site visitor data.

**Figure 7:**
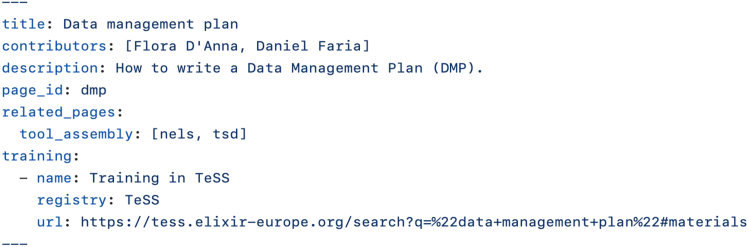
Front matter of the RDMkit “Data management plan” page

RDMkit page content is in Markdown, a user-friendly text markup language. Content is accompanied by page metadata specified in a dedicated section, the so-called “front matter,” of the markdown file illustrated in Figure 7. Page metadata play several key roles: (1) page IDs establish stable links between interconnected RDMkit pages and support tagging of tools and resources. (2) Metadata improve discoverability in web searches and enable structured linking within RDMkit and to external resources. (3) Including machine-actionable data, such as training links, enables a more interactive HTML presentation and supports centralized indexing—for example, to generate the “All training resources” page. Page metadata are provided by contributors and carefully reviewed by editors before final approval. Both contributors and editors must follow the correct syntax when linking to external resources, such as the TeSS training resource illustrated in Figure 7. Proper front matter syntax ensures these links appear appropriately in the “More information” segment of the page.

**Figure 8:**
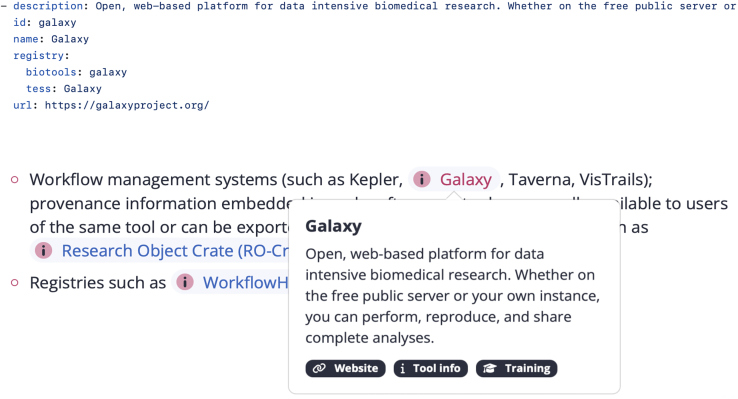
Managing the tools and resources list and displaying them in the RDMkit guidelines The top displays the entry for Galaxy in RDMkit’s “Tools and resources” YAML file. For each tool, RDMkit maintains an ID, name, description, and handles in registries like FAIRsharing, TeSS, and bio.tools. The bottom shows the snippet displayed for “Galaxy” in the “Data provenance” page where this tools gets mentioned. Tools are mentioned by their ID in the page source, which results in an interactive button that, when clicked, displays a snippet of information about the tool.

Site-wide data, such as tools and resource lists, page contributors, author affiliations, and featured events/news items, are stored as YAML files. The tools list serves as the database for all tools referenced in the RDMkit guidelines. An example YAML entry for the Galaxy platform^34^ and the visual display of the mentions of this tool in the RDMkit guidelines are given in Figure 8.

### Integrations with other resources

RDMkit follows two approaches to connect to other resources. These are bidirectional integrations with knowledge resources and unidirectional links to registries and external websites. The philosophy behind RDMkit integrations is to always link to information that is as specific as possible—depending on the context. RDMkit automates the maintenance of these links to ensure the freshness of the content. Links to external websites are hard-coded; however, they are automatically validated on a weekly basis, and broken links are raised as issues for site editors.

Knowledge resource integration requires content curation on both sides and, depending on the resource being connected, it may require development. This method was followed to integrate the FAIR Cookbook and the DSW. Thematic matching of RDMkit pages and FAIR Cookbook recipes is maintained as a YAML file in a dedicated GitHub repository. Both resources update their pages automatically based on this file. These automations also synchronize page titles and track new content with issue creation, enhancing sustainability and ensuring accurate cross-references between the platforms.

The DSW operates on hierarchical questionnaire structures, the so-called “knowledge models,” which allows models to be quite comprehensive. In line with RDMkit’s philosophy of “specific guidance,” DSW was extended with a “deep linking” feature, which allowed links from RDMkit pages to topic-specific questions nested in relevant DSW knowledge models. Linking from DSW to RDMkit was achieved by curating question hints with references to relevant RDMkit pages.

RDMkit’s preferred way of linking to registries is to point to specific registry entries or to thematic collections of entries. We were able to adopt this approach for links to FAIRsharing and bio.tools using unique identifiers of RDMkit-mentioned resources in those registries. Page authors are encouraged to provide resource registry identifiers; in case of missing identifiers, RDMkit will perform opportunistic link discovery by searching the registries with resource names in an automated fashion.

Training resources for a given RDMkit page topic are plenty. Therefore, TeSS links point not only to training collections, but also to training search views on TeSS. To support these views, RDM training was registered and annotated in TeSS using the EDAM^35^ ontology terms and the names of RDMkit pages as markup.

### Contribution and editorial processes

From the outset, we focused on building a community and participatory processes around the RDMkit. Community building started within the CONVERGE project^19^ and was extended to the ELIXIR Data Management Network.^18^ We also reached out to the ELIXIR communities and outside for contributions in specialized domains and fields of expertise. We identified the roles of the contributor, reviewer, and editor for participation. To ensure smooth communication within the community, we outlined a code of conduct on the website.

RDMkit infrastructure and core content were developed in virtual events. These events played a key role in community formation as they allowed the onboarding of various scientific domains and subject matter experts and led to the creation of most of the “Your domain” and “Tools assembly” pages. In addition to the events, we’ve created focus groups under the coordination of the ELIXIR Data Management Network. Focus groups work on longer timelines and create content on various topics of RDM, particularly in the sections “Your tasks,” “Your roles,” and “National resources.”

Contributing to RDMkit is designed to be open and easy. A dedicated website section was created to guide contributors in the contributing process. There are four ways to contribute: (1) using the GitHub GUI to edit page sources and suggest changes; editors assist by creating empty pages for new content; (2) for those unfamiliar with git, a Google Docs template is available, and editors transfer the content to the website; (3) site visitors can share comments and suggestions via a simple form on the website; and (4) experienced users can use the git command-line interface. Detailed instructions for each contribution method are available in the “Contribute” and “Editorial board guide” sections of RDMkit as well as in a style guide for text and design to help maintain consistency.

To maintain the quality, consistency, and coherence of the content, the editorial board reviews all RDMkit content. The editors oversee different sections, review content, identify gaps, and initiate contributions from experts. They follow the editorial board guide and checklist to ensure content aligns with RDMkit’s scope and style guide. Editors meet fortnightly to review and prioritize the work backlog using GitHub, and they proactively check on contributors. The editorial board also organizes virtual and face-to-face events, including annual editorial hackathons, to improve design, ensure consistency in style, and keep content relevant and up to date.

RDMkit contributors or readers can meet up with the RDMkit editorial board members in monthly RDMkit Club meetings. Topics such as new content needs, issues/questions related to RDMkit content, adoption of RDMkit for projects, and future directions of the RDMkit are discussed in these meetings.

## Resource availability

### Lead contact

Requests for further information and resources should be directed to and will be fulfilled by the lead contact, Carole Goble (carole.goble@manchester.ac.uk).

### Materials availability

This study did not generate new materials.

### Data and code availability

• All RDMkit content is licensed under CC-BY and is openly available at https://github.com/elixir-europe/rdmkit. RDMkit source code has been archived at Zenodo.^36^
• The RDMkit website is developed with the ETT, which is licensed under the MIT license. ETT source code is openly available at https://github.com/ELIXIR-Belgium/elixir-toolkit-theme. ETT source code has been archived at Zenodo.^37^

## Acknowledgments

The authors acknowledge the RDMkit community for their content contributions and the participating institutions and research infrastructures for their involvement in events and personnel support. We also thank Shoaib Sufi (https://orcid.org/0000-0001-6390-2616) for his review of the drafts of the paper. RDMkit was developed with funding from the European Union’s 10.13039/501100007601Horizon 2020 Research and Innovation Programme under grant agreement number 871075 as part of the ELIXIR CONVERGE project. RDMkit is supported by ELIXIR,^17^ the research infrastructure for life science data, and its ELIXIR Research Data Management (RDM) community.^18^ Current funding is provided through the ELIXIR Commissioned Service under the Project Plan for the RDM Community Implementation Study (Project ID: 2023-COMM-DATAREX, DATAREX – Data Management for Research in ELIXIR). The national ELIXIR nodes of Belgium, Norway, Sweden, and the UK have included the RDMkit content and infrastructure in their service delivery plans. ELIXIR Belgium: supported by Research Foundation – Flanders (10.13039/501100003130FWO) grants numbers I002819N and I000323N. ELIXIR Sweden: supported by funding from 10.13039/501100009252SciLifeLab, the 10.13039/501100004359Swedish Research Council (VR), the 10.13039/501100004063Knut and Alice Wallenberg Foundation, and the NBIS consortium. ELIXIR Norway: supported by the 10.13039/501100005416Research Council of Norway (Forskningsradet) through grants numbers 322392 and 295932. ELIXIR UK: supported by EOSC4Cancer funded through 10.13039/501100006041Innovate UK grant number 10038961 from the UKRI Horizon Europe guarantee.

## Author contributions

Authors contributions are listed as per Contributor Roles Taxonomy (https://credit.niso.org). P.A., F.D., B.D., and M.A., conceptualization, methodology, data curation, writing – original draft, writing – review & editing; B.D., software, validation; F.B., K.B., I.C., M.C., D.F., N.F., R.H., N.J., M.J., L.P.-S., U.W., D.P., G.P.-O., and M.P., conceptualization, methodology, data curation, writing – original draft; J.S. and M.S., software, methodology, writing – review & editing; D.W., R.A.B., and C.v.G., methodology, data curation, writing – review & editing; C.G. and F.C., conceptualization, methodology, supervision, project administration, writing – review & editing.

## Declaration of interests

The authors declare that they have no competing interests.

## Declaration of generative AI and AI-assisted technologies in the writing process

During the preparation of this work, the authors used Microsoft Copilot to check grammar, correct typos, and enhance the clarity and conciseness of the text. All content generated with the assistance of this tool was subsequently reviewed and edited by the authors, who take full responsibility for the final version of the publication.
