# RDMkit: A research data management toolkit for life sciences

**DOI:** 10.1016/j.patter.2026.101687

**Published:** 2026-09-09

**Authors:** Pinar Alper, Flora D’Anna, Bert Droesbeke, Munazah Andrabi, Rafael Andrade Buono, Federico Bianchini, Korbinian Bösl, Ishwar Chandramouliswaran, Martin Cook, Daniel Faria, Nazeefa Fatima, Rob Hooft, Niclas Jareborg, Mijke Jetten, Diana Pilvar, Gil Poiares-Oliveira, Marina Popleteeva, Laura Portell-Silva, Jan Slifka, Marek Suchánek, Celia van Gelder, Danielle Welter, Ulrike Wittig, Frederik Coppens, Carole Goble

## Main text

(Patterns *6*, 101345; September 12, 2025)

The author list for this article as originally published included incorrect affiliation information and misspelled the surname of author Gil Poiares-Oliveira. These errors have now been corrected in the article’s author list; the authors apologize for any confusion.

